# Clinical results of arthroscopic surgery in patients over 50 years of age—what viability does it have as a joint preservative surgery?

**DOI:** 10.1186/s13018-016-0504-9

**Published:** 2017-01-06

**Authors:** Mitsutoshi Moriya, Kensuke Fukushima, Katsufumi Uchiyama, Naonobu Takahira, Takeaki Yamamoto, Yojiro Minegishi, Masashi Takaso

**Affiliations:** 1 Department of Orthopaedic Surgery, School of Medicine, Kitasato University, 1-15-1 Kitasato, Minami-ku, Sagamihara, Kanagawa 252-0374 Japan; 2 Department of Rehabilitation, School of Allied Health Sciences, Kitasato University, Sagamihara, Japan

**Keywords:** Hip arthroscopy, Elderly patient, Joint preservation surgery, Osteoarthritis

## Abstract

**Background:**

To identify whether hip arthroscopy is a suitable option for treating hip pain in elderly patients and investigate the clinical outcomes of hip arthroscopic surgery for labrum tear and/or osteoarthritis in patients over 50 years of age.

**Methods:**

Between August 2009 and May 2014, a series of 23 patients (6 men and 17 women) with a mean age of 59 years underwent arthroscopy. We retrospectively examined the clinical records, radiographs, and outcome questionnaires from all patients. The mean follow-up period was 28 months.

**Results:**

The mean Japan Orthopedic Association hip score after surgery improved by a statistically significant amount. Eight patients (34.8%) were noted to have a progression of osteoarthritis (OA) diagnosed by radiograph, and one underwent THA after 13 months following arthroscopic surgery. The patients in which OA progression was noted were identified as having radiographical OA preoperatively and acetabular cartilage damage in the arthroscopic findings.

**Conclusions:**

Arthroscopic surgery performed in selected patients over 50 years of age might be beneficial if classified as Tönnis grade 0 preoperatively and/or classified as Outerbridge grade II in the arthroscopic findings.

## Background

Hip arthroscopic surgery is well known as a safe and less invasive alternative procedure for several hip disorders compared with conventional open surgery [1, 2]. In recent years, this surgery has been reported as having a high degree of usability in treating hip disease and has become a good option for treatment [3, 4]. However, existence of hip dysplasia and/or a high grade chondral injury have been reported as factors that indicate poor clinical outcomes following hip arthroscopic surgery [2, 5, 6, 7]. On the other hand, total hip arthroplasty (THA) has been recognized as a viable treatment solution for various hip disorders. Currently, the question remains if THA is the best treatment option in cases involving elderly patients with hip pathologies. Although THA has a great advantage in terms of early rehabilitation, there are some complications, such as durability and dislocation [8]. Additionally, patients who have a THA must restrict their sports activity [9]. We have attempted to indicate hip arthroscopic surgery as a joint preservative surgery after obtaining detailed informed consent for patients who have hip pain reported to be from a labral tear primarily (i.e., those with a symptomatic catching and positive anterior impingement test) and athletic performance difficulty, even though the patients were older, and low grade hip osteoarthritis was noted.

Here, the purpose of this study was to investigate the clinical results and the factors that indicate poor outcomes following hip arthroscopic surgery in elderly patients.

## Methods

Ethical approval from our Institutional Review Board was obtained for this study (approval number B16-32). Our indication criteria for hip arthroscopic surgery are as follows: (1) persistent groin pain and obtained informed consent; (2) abnormal physiological findings and abnormal lesion in the joint on detailed image inspections (e.g., labral tear, chondral damage) with using MRI and/or CT arthrography; (3) refractoriness to conservative treatment (i.e., medication and physical therapy) at least for 3 months; and (4) positive result of intra-articular procaine injection. In addition, we think as contraindication for hip arthroscopy to be acetabular dysplasia and osteoarthritis (OA). We obtained more careful informed consent in the cases which were thought as contraindication. Between August 2009 and May 2014, 134 hips in 128 patients who underwent hip arthroscopic surgery in our institute were investigated, retrospectively. For all patients, age, sex, and clinical history were assessed. In this study, our exclusion criteria were those less than 50 years of age and/or 1 year postsurgery as well as those who were diagnosed with inflammatory disease such as rheumatoid arthritis and purulent arthritis, and the case that diagnosed as synovial chondromatosis. After exclusion, the remaining 23 hips in 23 patients were included in this study (6 men, 6 hips; 17 women, 17 hips) with the mean age at the time of surgery 59.3 years (range, 53–75 years). The mean duration of follow-up was 28 months (range, 12–70 months). The patients were diagnosed clinically from the assessment of preoperative clinical findings (i.e., physical findings and radiographic findings) and an evaluation by using hip arthroscopy.

We retrospectively examined the radiographs, clinical records, and patient-reported questionnaires from all patients to assess the postoperative outcome. With regard to the radiological examination, all cases were investigated from anteroposterior (AP) and cross-lateral plain radiographs. The lateral centre-edge (LCE) angle of the hip joint was measured using an AP plain radiograph. A LCE angle < 20° was used to define a dysplastic hip. The presence of osteoarthritis (OA) was evaluated according to the Tönnis grading system (0: no signs of osteoarthritis, 1: increased sclerosis of the head and acetabulum, slight narrowing of the joint space, and slight lipping at the joint margins, 2: small cysts in the head or acetabulum, increasing narrowing of the joint space, and moderate loss of sphericity of the head, 3: large cysts in the head or acetabulum, severe narrowing or obliteration of the joint space, and severe deformity of the head) [10]. All radiological assessments were independently performed by two observers (MM and KF). The mean LCE angle value as calculated by each reviewer was recorded. In cases of disagreement regarding OA grade, the observers reviewed the radiographs together and conferred until agreement was reached. Clinical results were evaluated using the Japanese Orthopaedic Association hip score (JOA H-S). The hip score before surgery and the time of the final follow-up were assessed. The JOA H-S ranges from 0 to 100 points with points assigned as follows: pain, 0–40 points; range of motion, 0–20 points; walking ability, 0–20 points; and daily activity, 0–20 points [11]. The JOA H-S was recorded routinely by attending physician at each time, we investigated from clinical record. In cases that required THA after the hip arthroscopic surgery, the hip score was evaluated just prior to THA surgery. To evaluate quality of life (QOL) in patients with hip disease, the Japanese Orthopaedic Association hip disease eva1uation questionnaire (JHEQ) was used [12]. This questionnaire consists of three subscales: pain, movement, and mental, which are each 28 points. The maximum total score for all subscales combined is 84 points. In addition, dissatisfaction with the patient’s current condition and hip joint pain on each side are marked on a visual analog scale (VAS) from 0 mm (complete satisfaction or no pain at all) to 100 mm (complete dissatisfaction or maximum pain). Furthermore, perioperative surgical complications were investigated from clinical record.

### Arthroscopic assessment

Initially, the patient was placed in the supine position on a fracture table. Two portals (anterolateral and mid-anterior) were used in all cases. Gentle traction was applied to both legs, and a spinal needle was used to establish the anterolateral portal over a guide wire using fluoroscopy. With the camera in the anterolateral portal, the spinal needle was introduced into the location of the mid-anterior portal. Using these two portals, we assessed circumferentially the incidence of any labral tear and subsequent instability and classified any acetabular chondral injury by using the Outerbridge classification [13]. The grades are classified as follows: normal cartilage, grade I determined by softening and swelling, grade II determined by a partial-thickness defect with fissures on the surface that do not reach subchondral bone or exceed 1.5 cm in diameter, grade III determined by fissuring to the level of subchondral bone in an area with a diameter more than 1.5 cm, and grade IV determined by exposed subchondral bone. In cases in which labral instability was identified intraoperatively, we performed labral fixation using suture anchors. Furthermore, cases in which morphological abnormality related to femoroacetabular impingement (FAI) was identified in postoperative radiograph and impingement confirmed intraoperatively, we performed femoral cam osteochondroplasty. All patients were permitted to move to a wheelchair the day after surgery. For patients receiving labral fixation, partial weight bearing was permitted 1 week postoperatively, and full weight bearing 4 weeks postoperatively. For patients not receiving labral fixation, full weight bearing was permitted 1 week postoperatively. Radiographs were evaluated regarding whether there was progression of OA at the time of the last follow-up. In addition, we utilized a sub-analysis to evaluate the following factors influencing OA progression at the time of surgery: (1) age, (2) body mass index (BMI), (3) LCE angle, (4) radiographical OA grading, and (5) arthroscopic findings and treatments.

### Statistical analyses

Statistical analyses were performed by using JMP version 11.0 software (SAS Institute, Cary, NC). Results are expressed as the mean and the standard error of the mean unless otherwise indicated. A nonparametric Mann–Whitney *U* test was used for comparisons of normally distributed data among the groups (JOA H-S). A *P* value < 0.05 was chosen to indicate statistical significance.

## Results

### Clinical assessment

According to preoperative measurement of the LCE angle with plain radiographs, 1 hip (4%) had an LCE angle < 20°, 3 hips (13%) had an LCE angle between 20 and 25°, and 19 hips (83%) had an LCE angle ≧ 25°. According to preoperative evaluation of the radiograph with the Tönnis grading system, 15 hips (66%) found to be at grade 0, 7 hips (30%) at grade 1, and 1 hip (4%) at stage 2 were included. 5 hips (21%) were noted radiographical findings related to FAI (i.e., pistol grip deformity, crossover sign, excessive acetabular overcoverage).

The clinical results displayed a significant increase in the JOA H-S from a preoperative average from 76.7 ± 2.34 (range, 60–91) to 85.3 ± 3.02 (range, 51–100) at the time of the final follow-up (Table 1). Pain score and total score were also significantly increased. The JHEQ at the final follow up was 60.0 (pain: 20.0, motion: 19.6, mental: 20.4), and the mean postoperative VAS score was 32.3 ± 5.82 (range, 4–89). Perioperative surgical complications included one case of transient pudendal nerve palsy. The case was of a 55-year-old man with a septic arthritis, in whom erectile dysfunction (ED) occurred in the immediate postoperative period after 90 min of traction time. After 1 year of persistent ED, he was treated with medication by a urologist.

**Table 1:** Japanese Orthopaedic Association hip score results

Scoring items	Preoperative score	Score at last follow-up
Pain (0–40)	25.4 ± 1.4	31.3 ± 1.9^a^
ROM (0–20)	18.1 ± 0.5	19.3 ± 0.3
Ability to walk (0–20)	15.9 ± 0.6	16.5 ± 0.6
ADL (0–20)	17.3 ± 0.6	18.3 ± 0.5
Total	76.7 ± 2.3	85.3 ± 3.0^a^

^a^Statistically significant (*P* < 0.05)

### Arthroscopic assessment

Labral tears were identified in all hips. Chondral injuries classified using the Outerbridge system were assessed, and relatively worse degrees were found compared with that of the Tönnis-graded OA in preoperative radiographs (Table 2). The following 47.8% (11 cases) of Tönnis grade 0 or 1 were classified as grade 3 or more according to the Outerbridge classification. A labrum suture to manage labral instability was performed in 6 hips (26%). Osteochondroplasty at the femoral side to manage morphological deformity related to FAI (i.e., cam deformity) was performed in 3 hips (13%).

**Table 2:** Relationship of Tönnis grade and Outerbridge classification in our cases

Outerbridge classification		I	II	III	IV	Total
Tönnis classification	0	5	5	4	1	15 (65.2%)
	I		1	4	2	7 (30.4%)
	II				1	1 (4.35%)
		5 (21.7%)	6 (26.1%)	8 (34.8%)	4 (17.4%)	23

### Cases with progressing OA

Eight hips (35%) showed a difference in progression of OA after surgery. There was no correlation between the patients’ age, BMI, and progression of OA (Table 3). LCE angle was significantly greater in the maintenance group than in the progressive OA group (Table 3). The correlation of progressive OA and preoperative Tönnis staging is shown in Table 4. Though there were a few cases that showed progressive OA in Tönnis grade 0, many cases displayed a progression of OA with grade 1. The correlation of progressive OA and acetabular cartilage damage (as determined with arthroscopy) is shown in Table 5. All cases with OA progression were graded with III or more cartilage damage with the Outerbridge classification. Four hips that underwent suturing of the labrum showed a progression of OA.

**Table 3:** Comparison of progressive OA group with maintenance OA group

	Progressive group (8 hips)	Maintenance group (15 hips)	*P* value
Mean age	58.8. ± 2.9	59.6 ± 1.7	0.79
Mean BMI	24.2 ± 1.1	23.2 ± 0.9	0.57
Mean LCE angle	27.4 ± 2.7	34.1 ± 1.8a	0.04^a^
Diagnosis			
OA	6	3	
Labrum tear	2	10	
Septic arthritis	0	2	
Osteochondroplasty	0	3	

*BMI* body mass index, *LCE* lateral center edge, *OA* osteoarthritis

^a^Statistically significant (*P* < 0.05)

**Table 4:** Correlation between Tönnis classification and OA progression

Tönnis classification	OA progression (8 hips)
Grade 0	3/15 (20%)
1	4/7 (57.1%)
2	1/1 (100%)

**Table 5:** Correlation between arthroscopic findings and OA progression

		OA progression (8 hips)
Outerbridge classification	Grade 0	0
	I	0
	II	0
	III	IV
	IV	IV
Labrum tear	+	IV
	−	IV

## Discussion

In this study, we investigated the clinical outcomes of arthroscopic surgery for treatment of labrum tear and/or OA in patients over 50 years of age. Reports involving hip arthroscopy for the elderly have been published in recent years (Table 6) [1, 3, 14, 15]. Overall, although the clinical outcomes generally improved, they contained cases in which conversion to THA occurred at a constant rate. Malviya et al. reported that patients aged 50 years or older had a 4.65 times higher risk of requiring hip replacement compared to patients younger than 50 years in a large series of 6395 cases of hip arthroscopy [16]. On the other hand, Domb et al. performed a matching comparison test of hip arthroscopy care for patients > 50 years old and < 30 years old with a Tönnis grade of 0 or 1. Since there was no significant difference between groups, they concluded that hip arthroscopy for the elderly patient is a carefully selected intervention [1]. In the current study, the clinical assessment of JOA H-S and JHEQ showed satisfactory results especially in terms of pain relief. However, in 34.8% of the patients, a progression of OA was noted. As factors of OA progressed after arthroscopic surgery, Philippon et al. reported there was a significant difference in joint salvage rate when the preoperative joint space was less than or greater than 2 mm [3]. Redmond et al. reported a particular feature in cases that were converted to THA involving a lack of strong preoperative hip pain or dysplastic acetabular formation and intraoperative damage of articular cartilage [14]. In our study, all cases of grade III or more in Outerbridge classification exhibited OA progression after surgery as in the previous reports. Remarkably, 11 hips (47.8%), which were assessed as Tönnis 0 and 1 grades, were graded III or more using the Outerbridge classification during hip arthroscopic assessment. Fujii et al. examined intraarticular pathology with symptomatic developmental dysplasia during hip arthroscopy and reported that cartilage degeneration was recognized in 77.8% of those in the pre-arthritic stage [17]. Regarding arthroscopic findings in the current study, all cases of grade III or more in the Outerbridge classification showed OA progression after surgery. In addition, one of them was converted to THA. From the results, preoperative quantitative evaluation of articular cartilage was deemed important. Ellermann et al. reported on T2 mapping MRI for patients diagnosed with FAI to evaluate acetabular cartilage, and that it demonstrated accurately the difference between damaged and normal cartilage, comparable to the arthroscopic findings [18]. In the future, quantitative assessment of cartilage with MRI for preoperative evaluation might be helpful to determine the appropriateness of arthroscopic management.

**Table 6:** Reports about hip arthroscopy for elderly patients

	No.	Mean age (candidate)	Mean follow-up	Clinical result (pre-op → post-op)	Converted THA (rate, duration mean)
Javed et al. [14]	40	65 (> 60)	2.5	mHHS (60.5 → 79.5)	7 (17%, 1 year)
Philippon et al. [5]	153	57 (> 50)	5	mHHS (56 → 84)	31 (20%, 1.6 years)
Redmond et al. [13]	30	61 (> 60)	2.5	mHHS (63 → 80.1)	9 (30%, 1.1 years)
Domb et al. [1]	52	55 (> 50)	3	mHHS (60.5 → 84.2)	9 (17%, not listed)
Current study	23	59.3 (> 50)	2.8	JOA hip score (76.7 → 85.3)	1 (3.4%, 1 year)

*mHHS* modified Harris Hip Score

In terms of early postoperative pain relief and rehabilitation for elderly patients who experience hip pain, THA may be advantageous. However, although THA has longevity, there is a risk of complications such as aseptic loosening and/or dislocation [8, 19]. Wang et al. reported on the surgical outcome of THA in patients with osteonecrosis of the femoral head. In the Wang et al. report, specifically in elderly patients, 8.4% (9/107 hips) experienced postoperative complications including stem loosening, liner wearing, postoperative infections, postoperative dislocations, and pulmonary embolism [20]. We believed their study population was close to the population in early stage OA. Eitzen et al. reported on differences in gait characteristics in individuals with early stage hip OA who underwent THA and those who did not undergo THA. In their study, the individuals who did not undergo THA exhibited no decline in gait characteristics, minimum joint space, or overall function. Furthermore, their self-reported pain had significantly decreased [21].

How do we care for pain associated with early hip osteoarthritis? Teirlink et al. reported about physical therapy carried out in a multi-center pragmatic randomized controlled trial for patients with hip OA. There was no difference at the 12-month follow-up regarding function, although a difference was seen at the 3-month follow-up [22]. With regard to treatment with medication, non-steroidal anti-inflammatory drugs (NSAIDs) are generally used in the treatment of OA. Baigent et al. reported about the side effects of NSAIDs with a meta-analysis, including selective COX-2 inhibitors (coxibs) and traditional NSAIDs, and stated that all NSAID regimens increased upper gastrointestinal complications compared to placebo [23]. Emkey et al. reported that the pain relief rate was significantly lower for the participants treated with tramadol/acetaminophen in combination with a coxib, as compared with participants treated with placebo and a COX-2 NSAID [24]. However, in this multicenter, placebo-controlled study intended for OA patients, tramadol recipients frequently had complications of the central nervous system [25]. Drivers using tramadol aged 65 or older as included in this study were at a significantly increased risk of motor vehicle collision (odds ratio, 11.41) [26]. Consequently, an easy method of administration should be used and increasing the dose of the medicine should be avoided as much as possible.

Hip osteotomy surgeries appear to offer a good solution regarding joint preservation. Yasunaga et al. reported on rotational acetabular osteotomy in patients with advanced osteoarthritis secondary to developmental dysplasia of the hip, and Kaplan-Meier survivorship analysis, with radiographic signs of progression of osteoarthritis as the end point, and predicted a ten-year survival rate of 72.2% [27]. Teratani et al. reported the clinical and radiographic results of curved periacetabular osteotomy in patients ≥ 50 years of age compared to patients < 50 years old. Satisfactory results were seen in all radiographic measurements between the two groups preoperatively or postoperatively without significant difference [28]. However, due to the acetabular and/or femur deformities, surgical procedures are difficult with respect to the conversion to THA in cases where prior hip osteotomy was performed. Ohnishi et al. reported intraoperative fracture of 4 hips (6%) as a post-operative complication of THA osteotomy following hip osteotomy in 64 cases [29]. While some papers have also reported cases of conversion to THA after arthroscopic surgery, they did not show any complications with the procedure [30, 31]. In this study, one patient was converted to THA without issue during surgery. The same procedure seems to occur regarding THA after arthroscopic surgery and primary THA. Hip arthroscopy also has an advantage as a joint preservation surgery assuming a THA conversion.

With arthroscopy as an alternative to osteotomy and THA, the frequency of its complications should be mentioned. Gupta et al. investigated the incidence of complications for primary hip arthroscopy using a systematic review of 81 studies (5535 patients and 6277 hips) and reported low rates of major (0.41%) and minor (4.1%) complications [32]. The complication rate in our series was 4.3% (1/23) and was comparable with previous reports. Ultimately, hip arthroscopic surgery for elderly patients seems to be a less invasive treatment method compared to the other treatments mentioned previously. Cook et al. investigated the incidence of and risk factors for periprosthetic fractures following primary arthroplasty in 6458 cases, with the incidence of fracture 0.8% at 5 years and 3.5% at 10 years after the primary implant. Patients older than 70 and 80 years had a 2.9 and 4.4 times greater risk, respectively, of sustaining a subsequent fracture [33]. Therefore, it seems undesirable to perform THA in the early stages of OA.

Several limitations of the current study warrant mention. First, as a major limitation, sample size was too small. Second, there was lack of long-term result, lack of comparative group. Future studies should utilize a comparative evaluation with a larger sample size to clarify the effectiveness of hip arthroscopic surgery for elderly patients.

## Conclusion

Hip arthroscopic surgery might be a good option for pain relief, even in elderly people. This research indicates the feasibility of the use of arthroscopic surgery over conventional open surgery in this population.
